# Nigeria, a high burden state of obstetric fistula: a contextual analysis of key drivers

**DOI:** 10.11604/pamj.2020.36.22.22204

**Published:** 2020-05-18

**Authors:** Oluwasomidoyin Olukemi Bello, Imran Oludare Morhason-Bello, Oladosu Akanbi Ojengbede

**Affiliations:** 1 Department of Obstetrics and Gynaecology, College of Medicine, University College Hospital, University of Ibadan, Ibadan, Nigeria; 2 Centre for Population and Reproductive Health, Ibadan, Nigeria

**Keywords:** Obstetric fistula, burden, Nigeria, key drivers

## Abstract

Obstetric fistula (OF) remain a source of public health concern and one of the most devastating maternal morbidities afflicting about two million women, mostly in developing countries. It is still prevalent in Nigeria due to the existence of socio-cultural beliefs/practices, socio-economic state and poor health facilities. The country's estimated annual 40,000 pregnancy-related deaths account for about 14% of the global maternal mortality, placing it among the top 10 most dangerous countries in the world for a woman to give birth. However, maternal morbidities including OF account for 20 to 30 times the number of maternal mortalities. This review substantiates why OF is yet to be eliminated in Nigeria as one of the countries with the largest burden of obstetric fistula. There is need for coordinated response to prevent and eliminate this morbidity via political commitment, implementation of evidence-based policy and execution of prevention programs.

## Introduction

Obstetric fistula (OF) is an "abnormal opening between a woman's vagina and bladder and/or rectum through which urine (vesico-vaginal fistula) and/or faeces (recto-vaginal fistula) continually leak". This condition has devastating effects on a woman's life [1]. OF remain a source of public health concern to the United Nations and its member state because of the large number of women, (about 2 to 3.5 million) afflicted by it, mostly in developing countries including Nigeria with over 1 million women affected [1, 2]. Currently, there is no satisfaction in the rate of prevention and treatment of the backlog of obstetric fistula in Nigeria. Therefore, this review proposed to address the question "why OF is still persistent in Nigeria" and determine the reasons for the delay eradication progression which is crucial to optimizing the preventive approaches and treatment sustainability of obstetric fistula.

## Methods

The review was conducted using electronic literature search in PubMed, google, google scholar and Scopus database. This involved the use of search terms - obstetric fistula in Nigeria, obstetric fistula in sub-Saharan Africa, risks factors, burden of obstetric fistula, emergency obstetrics care (EmOC) in Nigeria, enablers or barriers to seeking fistula care and psychosocial impact of obstetric fistula. Abstracts of eligible articles were examined for relevance and appraised. Full text articles that provide information on the subject matter were fully evaluated. Also, the national strategic framework and elimination of fistula, Nigeria demographic and health survey(ies), international collaborators and NGO´s reports of those that have worked on fistula in Nigeria were reviewed.

## Current status of knowledge

**Figure 1:**
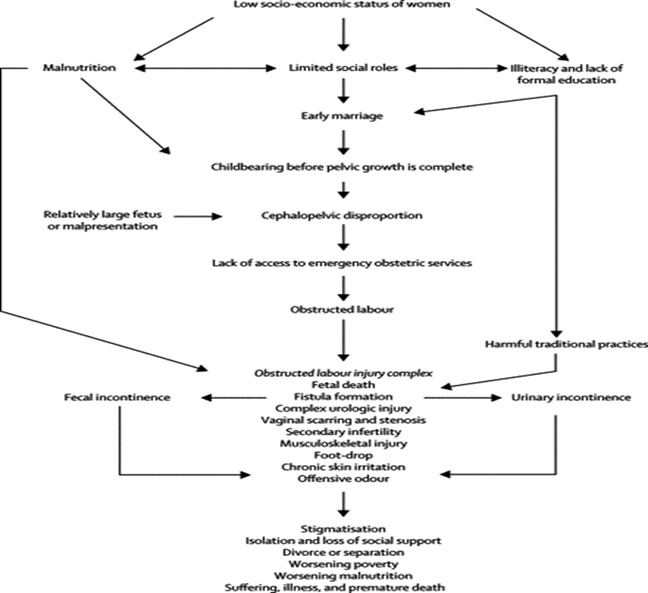
Risk factors of obstetric fistula

**Conceptual framework of pathogenesis of obstetric fistula:** OF is mainly caused by prolonged obstructed labour (POL); usually when the pressure of the baby´s head restricts blood flow and damages tissues between the vagina and the bladder or rectum. This condition is entrenched in poverty, predominantly affecting marginalized women who lack access to quality and EmOC, typically of lower socio-economic status, perform harmful traditional practices, with no or lower levels of education, dwelling in rural areas with preference for home delivery and avoidance of caesarean section, without prenatal care and married at younger age as shown in Figure 1 [3-5]. Recently, the insurgence of iatrogenic fistula following instrumental vaginal or caesarean delivery, hysterectomy for ruptured uterus or destructive operation by unskilled or poorly skilled personnel is of great concern [6, 7]. Meanwhile, lack of access to quality EmOC, has been recognized as the main underlying cause of fistula continuation in developing countries [8]. Several studies have identified different factors that contribute to the development of obstetric fistula which includes child marriage, unskilled traditional birth methods, unavailability of EmOC and poor antenatal care facilities [9, 10]. Additionally, poverty and malnutrition are considered as the root causes with the typical characteristics of obstetric fistula patient described as a young female, unbooked, poorly educated, short stature, who sustained trauma from first pregnancy and follows POL. It is worth noting that OF negatively affects the health of both the mother and the baby, as POL often results in stillbirth [10, 11].

**Epidemiological facts of obstetric fistula burden:** estimation of the burden of obstetric fistula in low-income countries is still a challenge [12]. However, lack of reliable data for prevalence and incidence has hampered efforts to formulate an appropriate and coordinated response to OF considering the challenges in the collection of accurate and comprehensive maternal morbidity data particularly for OF in low-income countries [2]. Approximately 2 million women were estimated to be living with unrepaired vesico-vaginal fistula (VVF) and about half of the total from developing countries were from Nigeria [13]. This number was considered to be an underestimation because the problem is believed to be widespread in Africa mainly and part of some Asian countries [14]. In Nigeria, prevalence of obstetric fistula is 3.2 per 1000 birth and it was estimated that about 13,000 new cases occur annually, suggesting that the backlog of unrepaired cases may take about 83 years to clear at the present rate of repair [7, 15]. Inclusively, United Nations International Children's Emergency Fund (UNICEF) reported that Nigerian women currently living with OF range from 400,000 to 800,000 and annually, an additional 50,000 to 100,000 new cases occur in the country [16]. This rising burden might be due to absence of reliable data for prevalence and incidence, accurate maternal morbidity data, commensurate human resources to address the backlog, poor funding and equipment to match this national burden. OF is considered one of the most serious and tragic childbirth injuries and often leads to depression, social isolation, chronic medical problems and deepening poverty [17, 18].

### Identified factors fueling the incidence of obstetric fistula in Nigeria

**Socio-economic factors:** socioeconomic characteristics of women have an impact on the risk of fistula and constitute major barriers to seeking care [18, 19]. OF extremely affects the underprivileged women, whose voices are scarcely heard. Studies have shown that OF predominately occurs among women with low economic status compared to their wealthy peers [19-22]. Likewise, living in rural areas put women more at risk of obstetric fistula, because not only are they marginalized in terms of health infrastructures, but they often live in remote areas, too far from health centres where they can receive timely EmOC [5, 23, 24]. Women affected with OF in Tanzania were reported to be predominantly farmers and these women will be 50% more economically impoverished by losing their job after sustaining the injury [5, 25]. Similarly, a prospective comparative case-control study in Northern Nigeria identified low socioeconomic status, transportation difficulties and rural place of residence as risk factors for OF [26].

**Nutrition:** malnutrition as a result of insufficient calcium and vitamin D could result in pelvic deformities or underdeveloped pelvis which predisposes women to cephalo-pelvic disproportion with resultant POL if EmOC is not instituted on time [27]. The Nigeria demographic health survey (NDHS) 2018 showed 12% of women of reproductive age (15-49 years) are underweight, with a body mass index of less than 18.5 and this has remained consistent at the same value for over a decade [28]. In addition, 1% of the women had short stature (<145cm) and this was reported to decrease with increasing level of education and wealth [28]. With consistent level of malnutrition and poverty among women of reproductive age, they are at greater risk of developing OF from POL due to inadequate pelvis coupled with a poor health seeking behavior as a result of impoverishment. Many of these women and girls who develop fistula find themselves trapped more in poverty and in most cases further malnourishment because they are excluded from community life, denied livelihood opportunities and abandoned by their husbands and families [29]. Although adequate nutrition is important in the prevention of OF, there are dearth of studies in this area despite its significance. However, improving women´s socio-economic condition will also improve girls/women´s nutritional status in order to eradicate malnutrition which could cause underdeveloped or deformed pelvis.

**Education/literacy:** education is constantly an essential part of any strategy on eradicating a disease of which OF is included. It does not only make the women and community knowledgeable; it also aids empowering women with resultant alleviation of poverty which has been documented as risk factors for OF. In Nigeria, education is a vital factor that contributes significantly in determining the age at marriage and first birth, educated females tend to marry later than uneducated ones [30, 31]. According to the NDHS, 2013, less than a quarter (11.5%) of girls aged 15-19 years completed secondary school while almost half (46.7%) of adolescent mothers with no education have begun childbearing or are pregnant with their first child [31]. Also, UNICEF reported that Nigeria has the highest number of dropout rate from school with one in every five of the world´s out-of-school children and about 16 million children especially girls aged 5-14 years been out-of-school [32]. This could be why Nigeria is one of the countries with the highest burden of obstetric fistula because maternal education has been reported to be a protective factor against the risk of obstetric fistula [33]. However, no Nigerian study was found to compare the educational status of women with OF with the general population but the largest population-based analysis of risk factors for vaginal fistula using 27 surveys from 23 countries in sub-Saharan Africa reported no significant direct effect of post-primary education on fistula occurrence [34]. Though, the extensive variability in the quality of education in sub-Saharan Africa suggests that women who develop fistula are the most disadvantaged of the underprivileged in a society [34]. Nonetheless, literate women have been reported to make better use of antenatal care, family planning information and other reproductive health services. There is also a correlation between mother's education and child nutrition with women of no or lower education having malnourished children and larger sized families [31, 35]. This will in turn lead to underdeveloped pelvis in the female children who are also likely to marry and start childbearing early thus are also at risk of obstetric fistula. Hence, increase female education in Nigeria will enhance empowerment and improve their access to quality antenatal care with more women delivering with skilled birth attendants thereby preventing POL which is the main cause of obstetric fistula. Additionally, female education could also lead to delayed marriages and childbearing with resultant growth into adulthood and well-developed pelvis thus preventing OF.

**Early marriage/childbearing:** early marriage is associated with maternal and infant morbidity(ies) since the woman would be biologically, economically and socially unprepared to cater for a family. Child marriage and childbearing at a young age contribute to the unacceptably high incidence of obstetric fistula. In Africa, 42% of girls are married before the age of 18 and Nigeria has the highest child bride population in the world with 23 million girls and women married as children which invariably leads to sexual activity for a girl at an age when she is neither physically nor sexually mature [36]. These young brides become pregnant at an early age and are more likely to die in childbirth or experience OF relative to those who get married later in life [36, 37]. Over the last three decades in Nigeria, data showed a slight decline of about 1% in child marriage per year and at this pace, the total number of child brides is expected to double by 2050 [37]. In the NDHS, 2018, 43% of women married before 18 years and about a fifth (19%) of the adolescent women age 15-19 years are already mothers or pregnant with their first child. The highest young motherhood was reported in the northwestern zone where OF is also high [28]. A study in Northwest Nigeria reported that almost all the married female adolescent interviewed are aware of fistula cases among their peers, had their marriage arranged by their father and that marriage timing is dictated by tradition and religion [38]. Also, in Northeastern Nigeria, 83.8% of the women with OF developed it before the age of 15 years and 93.7% of them had obstructed labour with average age at marriage of 14 years [26]. These young women are not only faced with the horror of obstetric fistula but are disempowered and denied the right to make decisions on when to give birth, how many children to give birth to, how to give birth, and to practice child spacing at will which further predisposes them to recurrence of obstetric fistula in future [10]. Additionally, almost half of the young women with no education have begun childbearing, and majority of them were from the poorest households in the national survey [31]. This could result in poor maternal and fetal outcome which contributes to the high maternal and infant morbidity and mortality in Nigeria. Early marriage is not only peculiar to Nigeria as a developing country but occurs in majority of African societies as a cultural norm [39]. Parents seek to marry daughters off early to protect them against premarital sexual activity and unintended pregnancy. Since early marriage and early childbearing are strongly correlated in developing countries, young girls become pregnant right after marriage, potentially without full development of their pelvis, which may increase the risk of developing OF. Women´s autonomy is another determinant of obstetric fistula [5, 23]. However, there is reform and emphasis on reducing early marriage in Nigeria by enacting laws outlining minimum age for marriage. These laws have been endorsed, but it fails to prevent forced or arranged marriage of girls below the legal age with parental consent which shows that the implementation of such law is still far much behind [40].

**Harmful traditional/cultural practices:** some of the major harmful traditional practices (HTPs) practiced in Africa that relates to OF include female genital mutilation (FGM), early/child marriage and son preference have received global attention on account of their severe and adverse effect on the health and well-being of girls [41]. Numerous efforts have been put in place to either modify or eradicate these traditional practices, but preventive interventions are often met with distrust or hostility from the communities practicing them [42, 43]. FGM in different forms could result in fistula or impaired female genital tract which ultimately endangers the health of the mother during childbirth. In northern parts of the country, FGM accounts for 2-13% of vesicovaginal fistulas [38]. FGM are often performed in infancy but some - gishiri and zur-zur cuts are performed during labour by traditional birth attendants in Northern Nigeria in order to expand the pelvic outlet to relieve obstructed labour and aid delivery and 30.3% of women who had this procedure were reported to have developed OF [44]. The role of female education cannot be over-emphasized in preventing FGM and associated morbidities. It is more likely that an educated woman will not subject herself or her child for harmful practice such as FGM. Also, lack of autonomy has an impact on the time frame to seek care, because women need permission from their spouse, or even their in-laws to go to a hospital, which can delay emergency care.

**Psychosocial damage:** aside enduring the ordeal of obstructed labour, women with OF face significant psychosocial challenges [45, 46]. Low self-esteem, feelings of rejection, stress, anxiety, mental health dysfunctions and post-traumatic stress disorders, loss of dignity and self-worth, loss of sexual pleasure, depression and suicidal thoughts are some psychosocial consequences that can accompany this morbidity [11, 18, 47, 48]. Incontinence often results in women experiencing extensive social stigma and related mental health issues, with these women either marginalized by households and communities, or marginalizing themselves [1, 3, 29, 49]. Such isolation and stigma acts to decrease their chances of seeking treatment [8]. Women often feel unfit to live with the rest of their family members and isolate themselves or are isolated by their families and communities. In many cases, women with fistula are divorced by their husbands [17, 34]. Social exclusion and lack of recognition experienced by women during the time they suffer from fistula (varying from a few months to many years) leads to a diminished sense of self-worth [50]. Due to physical impairment, the stigma and myths associated with the condition, a number of such women end up living apart and without economic support from their husbands or families. These coupled with the demise of their babies (most women with OF resulting from POL have still birth or early neonatal death) results in psychological distress. In a study on psychological therapy in OF care prior and after surgical intervention, the proportion of obstetric fistula women with depression score of 4 and above on the mental health ill status reduced from 71.7% to 43.4% after successful repair. Also, the OF women with score of less than 4 increased from 28.3% to 56.6% [47]. Likewise, a significant reduction in those with very low self-esteem from 65.0% to 18.3% was observed. Suicidal ideation also reduced with a percentage of 15.0% to 0% in those with severe, 16.7% to 5.0% in moderate and 25.0% to 21.7% in mild and those without suicidal attempt increased from 43.3% to 73.3% [47].

**State of emergency obstetric care (EmOC):** access to EmOC services is a key element of the World Health Organization ‘Making Pregnancy Safe´ programme. The WHO, UNICEF and United Nations Population Fund (UNFPA) developed guidelines for monitoring the availability and use of EmOC services [51]. In Nigeria, the unmet need for EmOC is high and unevenly distributed with only a few health facilities providing the standard EmOC [15, 52-54]. This poor quality of care is made worse by limited knowledge and availability of partograph, inadequate staffing and lack of infrastructures with only 4% of public health facilities meeting the EmOC standards. Other documented reasons for the poor quality of EmOC includes lack of skills on the part of available personnel to perform critical EmOC function and excessive delays [54-56]. In addition, less than 2% of women delivered by caesarean section nationally which is far lower than the WHO recommendation [43]. This could imply that more women may possibly have needed to be delivered via caesarean section but were not performed due to the unmet need of EmOC and such women may have their labour complicated with OF. As indicated by De Bernis *et al*. access to EmOC remains the most effective measures to reduce obstetric morbidity and mortality [1]. Nigeria lacks sufficient functioning maternity centres and women often fail to utilize available maternity services; therefore, OF with its devastating impact will continue to occur if not strengthened.

**Access to basic obstetric care:** the components of basic EmOC (BEmOC) include treatment for sepsis, eclampsia, post-abortion care (PAC), administration of oxytocics, removal of placenta and assisted delivery while the comprehensive EmOC (CEmOC) include caesarean section and safe blood transfusions in addition to all the BEmOC functions [43, 54]. Majority of the primary healthcare facilities in Nigeria are unable to adequately provide basic EmOC services or meet an increasing demand for obstetric care. Only 43% of births were attended by skilled birth attendants and about two-third (67%) of pregnant women received antenatal care (ANC) at least once during pregnancy [28]. However, exploring the recommended minimum of four ANC visits makes Nigeria antenatal care coverage to be the third lowest in sub-Saharan Africa [57]. A marked decline was also found among the ANC services user when considering the inequalities in their socioeconomic and educational status with only 41% of women in the lowest wealth quintile and 45% of those with no education accessing ANC [28]. If a woman is not able to access the basic obstetric care - ANC, she might not benefit from preventive measures and EmOC which makes her more prone to maternal morbidities including OF and mortality. Lack of geographical and economic access to caesarean section services have been established to be an immediate cause of obstetric fistula in Nigeria [15]. Therefore, for a significant reduction in maternal morbidity and mortality rate in Nigeria, attention should be focused on increasing the number of facilities with EmOC capability, improving the quality of facilities and both identifying and addressing the barriers faced by Nigerian women in accessing these facilities [58].

**Trained traditional birth attendants (TBAs):** TBAs are community-based providers of care during pregnancy, childbirth and postnatal period without formal medical training and are traditionally independent of the health system [59, 60]. A review on delivery by unskilled birth attendant in Nigeria revealed that the prevalence of TBA-assisted delivery remained unchanged between 1999 and 2018 [60]. Usually, the TBAs are more accessible and available within the community. However, their inability to acquire life-saving skills, lack of supervision, lack of integration into the health care system and absence of emergency backup systems have been identified as reasons for their ineffectiveness [61-63]. Low maternal education, rural residence, poor family wealth index, unemployed status and having more than 5 living children are identified factors predicting delivery with TBAs in Nigeria [64]. Studies have reported that programmes focused on TBAs training failed to show a significant reduction in maternal mortality [63, 65]. To buttress this, the 1940s and 70s initiatives that excluded the TBAs have been shown to improve maternal health as seen in the reduction in maternal mortality in the diocese of Niger Eastern, Nigeria to about less than 50 deaths to 100,000 live births and elimination of obstetric fistula in the Zaria region respectively [66]. TBAs were documented to be mainly responsible for unbooked emergencies resulting in high death rate of 2900 per 100,000 births in Zaria due to their inability to treat principal causes of maternal deaths [66]. The adverse outcomes of maternal mortality, morbidity and disability that result from delivery by TBAs occur because they are unskilled in managing pregnancy and labour complications and lack skills for risk stratification [60]. Notwithstanding, the TBAs can still contribute to the reduction of maternal morbidity and mortality by facilitating facility and skilled attended births in the community but this can only be feasible if they are appropriately integrated with the local health system. They can also help to break socio-cultural barriers on intervention on reproductive health programmes.

**Making pregnancy safer initiative:** this was initially called safe motherhood initiative which is built on four pillars namely: family planning, antenatal care, clean safe delivery and essential obstetric care. These are series of initiatives, practices, protocols and service delivery guidelines designed, to ensure that women receive high-quality gynaecological, family planning, prenatal, delivery and postpartum care so as to achieve optimal health for the mother and fetus during pregnancy, childbirth and postpartum. Its practice is a key strategy for reducing maternal and infant morbidity and mortality. As at 2018, Nigeria´s maternal mortality ratio was estimated to be 512 deaths/100,000 live births [28]. Several studies identified long distance to health facility, onset of labour at night, unavailability or lack of money for transportation, unsatisfactory services at health facility, unfriendly attitude or unavailability of staff at health facility, lack of urgency at health facility and previous uneventful delivery as factors responsible for non-utilization of maternal health services [54, 67]. Poor utilization of health facilities during delivery by pregnant mothers is of great concern and still a major cause of maternal and newborn morbidity and mortality in Nigeria. However, significant determinants of the utilization of health care is largely dependent on education, mothers´ age, socioeconomic status and urban residence as reported in a study conducted in Ogun state and substantiated by the national survey [28, 68]. Therefore, fortifying maternal and newborn health efforts at all levels, that is global, regional, and national in the context of equity, poverty reduction and human rights will positively affect the utilization of maternal health services.

**Barriers to successful fistula repair in Nigeria:** every case of obstetric fistula can be prevented with timely access to EmOC, especially caesarean section. Currently, a large backlog of women requiring fistula repair experience long waits due to limited number of fistula surgeons even with the training of new ones because new cases of fistula occurs faster than the treatment of the existing ones [14]. Though, there is dearth of national data that have accessed the accurate reduction in the incidence and prevalence of obstetric fistula in spite of the fact that the NDHS, 2018 showed that the knowledge of fistula among women remained 31% over the last decades [28]. However, according to the analysis carried out by Amodu *et al*. it was observed that the Nigerian constitution, justice environment and the obstetric fistula policy, 2011-2015 do not reveal clear commitment to eradicating obstetric fistula [40]. There is an indication of continued inequity distribution of social resources for women, access to maternal health care and reproductive rights which together constitute fundamental factors in the cause of obstetric fistula. To this end, the National Strategic Framework for the Elimination of Obstetric Fistula was developed to increase access to prevention, treatment and rehabilitation services [4, 15].

**Way forward, call to action/elimination of obstetric fistula:** intensive efforts should be made by governmental and non-governmental organizations to reduce the incidence or eradicate OF through engagement of stakeholders, innovative strategy to improve access to family planning and policy support to prevent OF. Improved access and uptake of family planning will prevent pregnancy thus reduce the risk of developing OF. Also, there is need to ensure availability, affordability and accessibility of quality maternal health services including EmOC by strengthening the health care system [15]. Unceasing surveillance of women seeking fistula care is necessary to track the met need for surgical repair at sub regional, regional and national levels. In addition to the stand-alone fistula centres in states where OF is relatively common, maternity-care facilities should offer diagnosis and surgical repairs, particularly simple and uncomplicated fistula so as to be able to provide a continuum of care and reduce backlog. Other approaches should include training of care providers on the prevention and diagnosis of fistula during the antenatal, intrapartum, postpartum periods, early identification and treatment of iatrogenic fistula, prompt referral system as well as assessing all women with fistula-like symptoms. Emphasis should also be made on outreach and prevention services for rural and malnourished women, since they are at greatest risk. Ultimately, women and girls´ human rights must be upheld and protected by government at all levels to achieve equity of access and outcomes. To end obstetric fistula and fully realize the rights of women and girls, we must address the structural drivers that perpetuate inequalities.

**Training and capacity building - unlocking potentials:** in low-resource countries like Nigeria, FIGO (International Federation of Gynaecology and Obstetrics) provides education and training in building capacities to promote women´s health through good practice, strengthening of leadership, and promotion of policy dialogues [69]. They provide training at different levels on clinical management of fistula - standard, advance and expert level based on the severity of the fistula. In 2011, the FIGO and partners (ISOFS, UNFPA, Engender Health, Pan African Urological Surgeons Association, the Royal College of Obstetricians and Gynaecologists and the Fistula Foundation) published a training manual that uses a modular competency-based approach which is now the standardized manual for fistula care and management [70]. Since then, there has been training courses run in several countries with high burden of obstetric fistula which has gradually reduced the backlog of obstetric fistula. In Nigeria, the trainees are trained for a minimum of 6-8 weeks in an accredited facility for achievement of a standard level of competency [70]. Consequently, the FIGO and partners competency-based program has contributed to the delivery of good care in terms of fistula trainer-trainee program. Additionally, the training program is helping to deliver a consistent method to the medical and surgical management of fistula. Furthermore, an approach involving expert surgeons in tertiary hospital performing fistula repairs at secondary and primary level of healthcare has being yielding positive results in Nigeria. Clients are mobilized for pooled efforts which are fistula camps where large numbers of fistula are repaired. These serves as a fulcrum for training new and upgrading the skills of fistula surgeons while mopping up large numbers of existing cases. These approaches have been successful in different regions of the country and expanding such innovation will improve skills and increase the number of fistula surgeons available for repair.

**Sustainable investment for obstetric fistula elimination:** in Africa, especially the West and Central Africa there is scarcity of information on the cost of fistula surgeries as well as the quantitative impact on women´s earnings. On the other hand, UNFPA estimates the average cost of obstetric fistula repair to be around 500 dollars per woman [71]. Furthermore, the study on cost of fistula repair conducted in Nigeria and Ethiopia, recommended the adoption of the costing tool to provide cost estimates for direct costs associated with fistula care, hospitalization and transport [72]. The implementation and monitoring of the national strategic framework for the elimination of obstetric fistula should be ensured so as to achieve the main goal of 30% reduction in the backlog of untreated cases, the incidence of obstetric fistula, rehabilitation and reintegration of the repaired needy fistula patients by 2023 [15]. This will result in a framework that aligns the global vision of ending fistula by 2030 and will be consistent with achieving a Nigeria-free obstetric fistula.

## Conclusion

Nigeria has one of the largest burdens of obstetric fistula till date, it is imperative to organize a well-coordinated response of adequate investment in infrastructure, human capital and policy direction that will put the country on the pathway of a sustainable prevention and elimination of this vexing morbidity. Briefly, there should be political commitment, evidence-based policy, prevention programs against early marriage, FGM eradication and family planning, cheap and affordable interventions; emergency obstetric care and training of champions to lead advocacy, prevention, treatment and rehabilitation.

### What is known about this topic

- Nigeria has a high prevalence of obstetric fistula and it will take several years to clear the backlog of unrepaired cases;
- Currently, there is no satisfaction in the rate of prevention and treatment of the backlog of obstetric fistula in Nigeria;
- Existing literatures have established the risk factors, causes, symptoms, management and rehabilitation/reintegration of women with obstetric fistula.

### What this study adds

- This study has been able to identify why obstetric fistula is persistent in Nigeria despite the local and international efforts;
- This study provides reasons for the slow progress in eradication of obstetric fistula and drivers that perpetuate the inequalities;
- This study proffers laudable solutions on how to eliminate the condition in Nigeria.

## Competing interests

The authors declare no competing interests.
